# Phylogeography of the Tibetan hamster *Cricetulus kamensis* in response to uplift and environmental change in the Qinghai–Tibet Plateau

**DOI:** 10.1002/ece3.5301

**Published:** 2019-06-02

**Authors:** Li Ding, Jicheng Liao

**Affiliations:** ^1^ School of Life Sciences Lanzhou University Lanzhou China

**Keywords:** climatic change, *Cricetulus kamensis*, demographic history, phylogeography, Quaternary glaciations, species distribution model

## Abstract

**Aim:**

The evolutionary process of an organism provides valuable data toward an understanding of the Earth evolution history. To investigate the relationship between the uplift of the Qinghai–Tibet Plateau (QTP) and mammalian evolution since the late Cenozoic, the geographic distribution of genetic variations in the Tibetan hamster *Cricetulus kamensis* was investigated using phylogeographical methods. In particular, population divergence, demographic history, genetic variation, and the prediction of species distribution area were investigated.

**Location:**

The Qinghai–Tibet Plateau.

**Methods:**

A total of 53 specimens, representing 13 geographic populations, were collected from the QTP. The phylogeographical pattern and demographic history of *C. kamensis* were analyzed, and the probable factors in the QTP uplift and the Quaternary glacial periods were inferred from one nuclear and four mitochondrial genes. Furthermore, the species distribution model (SDM) was used to predict changes in potentially suitable habitats since the last Interglacial.

**Results:**

Phylogenetic analysis demonstrated that two major genetic differentiations of the *C. kamensis* population occurred during the Early Pleistocene that were influenced by the Qing‐Zang tectonic movement from the Middle Pliocene to the Early Pleistocene. Genetic distance between two major clades indicated low genetic divergence. Demographic history analysis showed that the *C. kamensis* population was affected by the Quaternary glacial period. SDM analysis indicated that *C. kamensis* was endemic to the QTP and the suitable habitat was affected by climate change, especially during the Last Glacial Maximum (LGM).

**Main conclusion:**

Our results indicated that the QTP uplift led to the population divergence of *C. kamensis*, and vicariance well accounted for the geographic distribution of genetic variation in *C. kamensis* as a result of genetic divergence and lack of gene flow. The genetic distance shows that *C. alticola* may be a subspecies of *C. kamensis*. Demographic history analysis suggests that the QTP was affected by the last glacial period. SDM analysis supports that almost the entire QTP is covered by a huge ice sheet during the LGM.

## 1 INTRODUCTION

Phylogeography is an integrative discipline that focuses on the principles and processes underlying the geographic distributions of genealogical lineages, namely those that clarify geological history and phylogenetic components of the spatial distributions of gene lineages (Avise, 2000). Molecular phylogenetic reconstructions of biotas can help to test biogeographic hypotheses, illustrate evolutionary relationships between spatial and temporal population structures of species, and support speculations of speciation mechanisms using genetic data across the distribution range of species under the phylogeographical approach. The Qinghai–Tibet Plateau (QTP) is a relatively young geological structure with a complicated evolutionary history, complex landform configuration, unique climatic environment, and diversified habitats (Favre et al., 2015; Lei, Qu, & Song, 2014). During recent years and by using a phylogeographical approach, a large number of studies indicated that the distribution patterns and population genetic structures of plateau‐dwelling organisms are related to the tectonic configuration and climate change in the QTP (Deng, Wang, Wang, Li, & Hou 2015; Favre et al., 2015; Lei et al., 2014; Yang, Dong, & Lei, 2009).

Historically, the uplift of the QTP happened in several steps and followed an asynchronous uplift with several partitioned blocks, showing a progressive uplift from south to north (Mulch & Chamberlain, 2006; Wang et al., 2008a). However, the rapid uplift during the late Cenozoic plays a major role in shaping the landform and climate of the QTP, as well as exerting a remarkable effect on the biota (An et al., 2006; Deng et al., 2015; Li & Fang, 1999; Shi, Li, & Li., 1998). Its process of geographic formation and the evolution of biotas remains insufficiently understood despite its outstanding geographic extent so far (Favre et al., 2015). Several recent studies have suggested that the uplifting of the QTP plays a major role in shaping plateau biodiversity due to strong ecological gradients in temperature, precipitation, and topography which contribute to the generation of a variety of terrestrial habitats (Deng et al., 2015; Favre et al., 2015; Lei et al., 2014). A large number of studies demonstrated that the phylogeny, speciation, and demography of organisms are associated with the uplift of the QTP and simultaneous changes of climate, for example in frogs, lizards, birds, and mammals (James et al., 2003; Jin, Brown, & Liu, 2008; Liu et al., 2015; Tseng et al., 2014). With respect to mammals, Yu et al. (1992) proposed that the diversification of pika correlates with the uplift of the QTP in China; furthermore, geographic isolation created numerous diversified microhabitats for pika survival. Molecular systematics of the genus *Ochotona* also indicate that the speciation of pikas is consistent with the historical episodes of geologic and climatic changes of the QTP (Niu, Wei, Ming, Liu, & Feng, 2004; Yu, Zheng, Zhang & Li, 2000); however, studies on the intraspecific divergence of pika on the QTP have not been reported. The population divergence (between 2.4 and 4.4 Mya) of the Tibetan gazelle (*Procapra picticaudata*) was influenced by the uplift of the QTP (Zhang & Jiang, 2006), whereas its dispersal capacity far exceeds that of muroid rodents. In addition, a similar result was reported in a study of the plateau zokor (*Eospalax baileyi*) (Tang et al., 2010). Its life history is completely different from the extant mice. The hamster is an ancient animal in comparison to the aforementioned mammals, and their fossil data can be traced back to the middle Eocene (approximately 40 Mya). The hamster has been considered to originate from Asia (Huang, 2004; Storer, 1988; Tong, 1992). Herein, the phylogeographical study of the hamster living on the QTP is more likely to reveal the geographic and climatic evolution of the QTP. Small mammals, such as rodents, are generally good models to test the relationships between geology and climatic events as well as for the study of the evolution and adaption of animals, such as their localized habitat requirements and low dispersal abilities. These animals are generally limited to persist on a particular site even under extreme environmental conditions (Nava‐García, Guerrero‐Enríquez, & Arellano, 2016; Wang, Zhao, Fang, Liao, & Liu, 2014). Given their tendency toward a short generation time and rapid mitochondrial DNA substitution, speciation seems to be their main strategy to adapt to the original environment despite habitat constraints (Ben Faleh et al., 2013).

The Tibetan hamster *Cricetulus kamensis* (Satunin, 1902) is endemic to the QTP and is a small to medium sized hamster, similar to *C. longicaudatus* with restricted distribution in the QTP (Feng, Cai, & Zheng, 1986; Luo et al., 2000). To date, there have been relatively few publications about *C. kamensis*, which might be the reason why this species is poorly known outside of China. The classification of *C. kamensis* has long been controversial. Historically, Tibetan hamster has been classified into four species (*U. kamensis*, *C. kozlovi*, *C. kama,* and *C. alticola*) from different biogeographic populations based on their respective morphological characteristics (Bonhote, 1905; Satunin, 1902; Thomas, 1917). As such, Smith and Xie (2013) persist in this view on Tibetan hamster classification. Argyropulo (1933) sorted the Tibetan hamster specimens and divided them into three species (*C. kamensis*, *C. kozlovi,* and *C. lama*), while Lebedev and Potapova (2008) pointed out that *C. kozlovi* is a subspecies of *C. migratorius* based on the pseudosciuromorphous structure of the zygomatic plate. Corbet (1978) admitted that both *C. kamensis* and *C. alticola* are valid species and other two species (*C. kozlovi* and *C. lama*) are synonymous of *C. kamensis*. Subsequent taxonomic publications on Tibetan hamster adopt the classification conclusion of two separate species (Don & DeeAnn, 2005; Lebedev et al., 2018). However, several studies on Tibetan hamster classification suggested three species (*C. kozlovi*, *C. lama* and *C. alticola*) are subspecies of *C. kamensis* rather than that of a separate species (Feng et al., 1986; Luo et al., 2000; Wang & Cheng, 1973; Zheng, 1979). In summary, the classification of Tibetan hamster has not been determined.

Prior phylogenetic analysis suggests that *C. kamensis* is the first species to differentiate among *Cricetulus* species with a long evolutionary history (Ding, Li, & Liao, 2016). This plateau‐dwelling rodent species inhabited high‐altitude environments for a long time, which is related to the geological history and climatic change of the QTP. Hence, this species can provide an ideal model organism for the study of the historical biogeography of this region. Herein, we hypothesized that the current distribution of *C. kamensis* was influenced by physical events (such as the tectonic configuration) and climatic change (such as cold, dry, and glacial periods) of the QTP. In the present study, one nuclear and four mitochondrial genes were used to (a) detect the relationship between the phylogeographical pattern of *C. kamensis* and the uplift of the QTP, (b) examine both the population genetic structure and demographic history of *C. kamensis*, (c) assess the likely cause for the decrease of habitats suitable for the survival of *C. kamensis* during the Late Pleistocene glacial period, and (d) discuss the taxonomical issue of *C. kamensis* by using phylogenetic analysis and genetic distance.

## 2 MATERIALS AND METHODS

### 2.1 Sample collection and ethics statement

**Figure 1:**
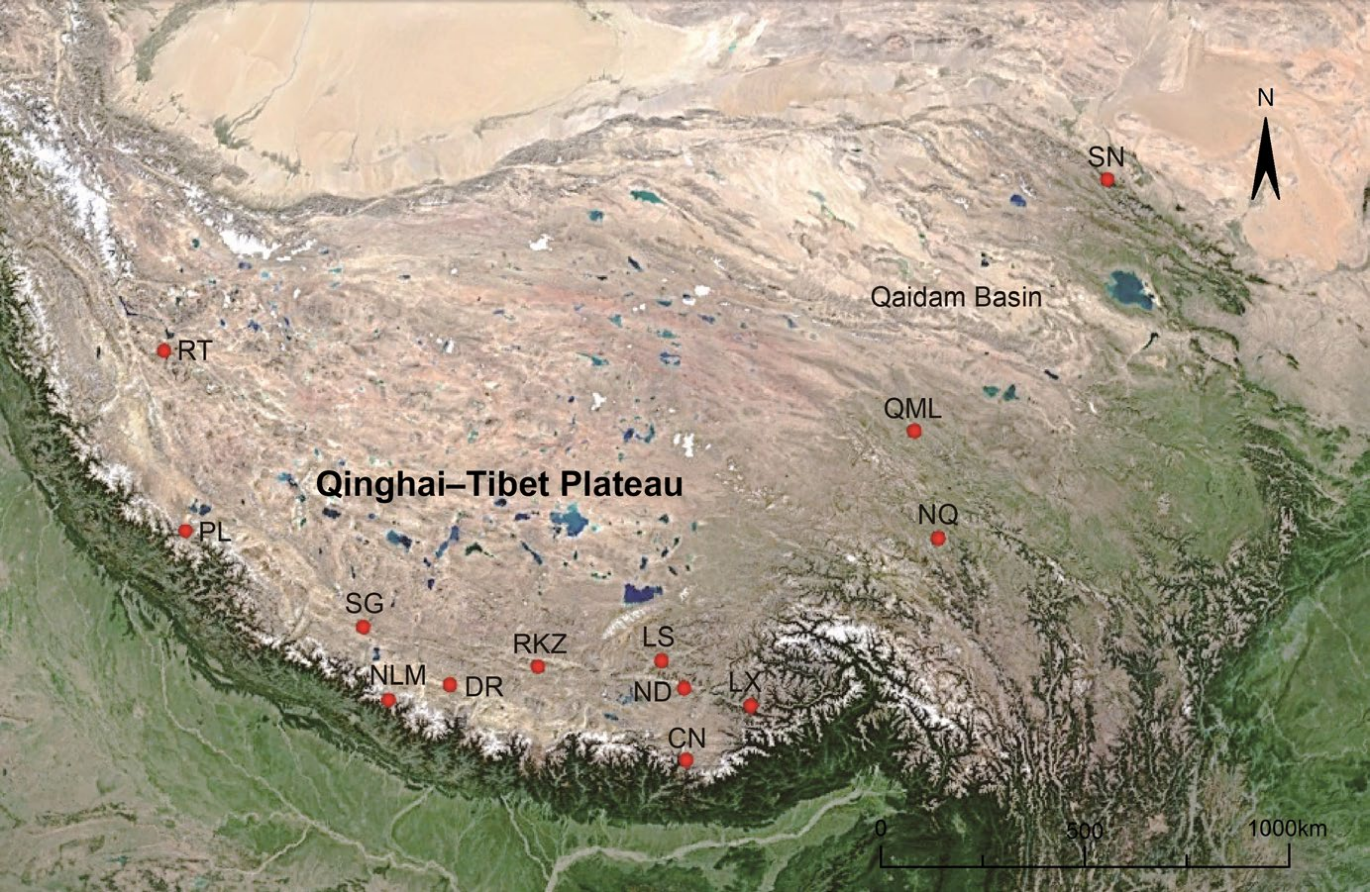
Localities of *C. kamensis* sampling sites. For the names of the localities, see Table 1

A total of 53 *C. kamensis* were trapped in the QTP area from 13 geographic locations (Table 1; Figure 1). The specimens were identified in the field based on external morphological characteristics (Feng et al., 1986; Luo et al., 2000). Muscle tissue of *C. kamensis* was collected from these individuals; then, both tissue and voucher specimens were preserved in absolute ethanol and formalin solutions, respectively. Neither sample collections nor the study conflicted with the ethical guidelines, religious beliefs, or legal requirements of China. All animal experiments conformed to the guidelines for the care and use of laboratory animals and were approved by the Committee of Laboratory Animal Experimentation at Lanzhou University (Lanzhou, China).

**Table 1:** Geographic population information of *C. kamensis* used in this study

Sample site	Code	Latitude (°N)/longitude (°E)	Altitude (m)	Sample size	H	UH	Hd	π	*K*
Burang	PL	30°15′11″/81°10′01″	3,890	4	4	4	1.000	0.001	3.667
Cona	CN	28°00′23″/91°57′55″	4,341	3	2	1	0.667	0.002	8.000
Lhasa	LS	29°39′47″/91°13′00″	3,646	2	1	0	0	0	0
Nang	LX	29°05′40″/93°05′03″	3,089	6	2	1	0.333	0.007	29.333
Nangqen	NQ	32°18′52″/96°27′03″	3,770	1	1	1	–	–	–
Nedong	ND	29°14′46″/91°43′59″	3,556	5	3	1	0.800	0.004	19.400
Nyalam	NLM	28°9′46″/85°59′10″	3,764	6	4	4	0.867	0.001	4.067
Qumarleb	QML	34°06′53″/95°44′35″	4,086	2	2	2	1.000	0.002	7.000
Rutog	RT	33°13′56″/79°46′56″	4,303	1	1	1	–	–	–
Saga	SG	29°19′24″/85°13′27″	4,488	2	2	2	1.000	0.006	26.000
Shigatse	RKZ	29°13′37″/88°49′07″	3,927	8	5	3	0.857	0.003	11.250
Sunan	SN	38°50′13″/99°36′56″	2,297	6	4	2	0.867	0.001	4.800
Tingri	DR	28°38′28″/87°08′44″	4,391	7	5	4	0.857	0.016	68.571

Abbreviations: π: nucleotide diversity; H: Number of haplotypes; Hd: haplotype diversity; K: average number of nucleotide differences; UH: number of unique haplotypes.

### 2.2 Laboratory protocols

The total genomic DNA was extracted from the muscle samples of *C. kamensis* using the TIANamp Genomic DNA Kit (Tiangen). Polymerase chain reaction (PCR) was used to amplify both mitochondrial DNA (mtDNA) and nuclear DNA (nuDNA) fragments of *C. kamensis*, in which four primer pairs were used to amplify four mtDNA markers (cytochrome c oxidase subunit 1 (Cox1), cytochrome c oxidase subunit 3 (Cox3), cytochrome b (CYTB), and control region (D‐loop), Table 2). Two primer pairs were used to amplify partial sequences of von Willebrand Factor exon 28 (vWF) using both standard PCR and nested PCR (Table 2) (Porter, Goodman, & Stanhope, 1996). Nested PCR was used to amplify vWF fragments that required two pairs of primers (outside and inside primer pairs), the outside primer of which was used to enrich the target region from complex genome and the inside primer was used to amplify the target region from the first round PCR products (Shen, Liang, Feng, Chen, & Zhang, 2013). The inside primer pair of vWF was specifically designed for *C. kamensis* based on other conservative hamster homologous sequences of vWF (Table 2). PCR was employed to amplify DNA fragments using a 25 μl reaction system that contained 2.5 μl 10 × PCR buffer, 1.5 μl 25 mM/L MgCl_2_, 0.5 μl 2.5 mM/L dNTP, 1 μl of each primer pairs (10 μM each), 0.5 μl Taq polymerase (5 U/μl, Sangon, Shanghai), 1 μl DNA template, and 17 μl ddH_2_O. The strand PCR was run with pre‐denaturing for 3 min at 95°C, followed by 35 cycles of denaturing for 30 s at 95°C, annealing for 45 s at 50–60°C, extension at 72°C, and a final extension at 72°C for 10 min. All PCR products were detected using 1% agarose gel electrophoresis and then directly sequenced using the Sanger sequencing method (Genewiz Biotech (Suzhou) Co., Ltd.). DNA sequences were manually edited and assembled using Lasergene version 5.0 (DNASTAR). Here, five DNA markers (Cox1, Cox3, CYTB, D‐loop, and vWF) have been deposited in the GenBank database and GenBank accession numbers please see the section of DATA ACCESSIBILITY4.1.

**Table 2:** Primer pairs for mtDNA and nuclear markers used in this study

Primer name	Sequence(5′–3′)	Expected size (bp)	Source
Cox1‐H	ACTACTGGCTTCAATCTACTTCTC	1,792	In this study
Cox1‐L	AAGACATAGAGGTTATGGAGTTGG		
Cox3‐H	CCACCAACCGCCATAATCAC	1,050	In this study
Cox3‐L	GGATTCAGAACGCAACTATGATAAG		
CYTB‐H	TAAGTGCCGCTCCCAAACAA	1,360	In this study
CYTB‐L	CTTCCTTCTTGATGCCCTGAG		
D‐loop‐H	ATCGGCCAACTCGGATCAATCATT	1,300	In this study
D‐loop‐L	GTGTGCTTGATACCGTCTCCTTGG		
Out‐vWF‐H	CTGTGATGGTGTCAACCTCACCTGTGAAGCCTG	1,300	(Porter et al., 1996)
Out‐vWF‐L	TCGGGGGAGCGTCTCAAAGTCCTGGATGA		
In‐vWF‐H	CTCCCGCATCACTATACTCCT	660	In this study
In‐vWF‐L	GTGACCATGTAGACCAGATTAGG		

### 2.3 Phylogenetic analysis

A total of 38 haplotypes (53 individuals) were used to build the phylogenetic relationships between 13 geographic populations. Each concatenated sequence had a length of 5,078 bp, including outgroup taxa. A concatenated sequence consisted of four mtDNA genes (Cox1 + Cox3 + CYTB + D‐loop) and one nuclear gene (vWF) fragment, and each marker was separately aligned and these sequences were then manually concatenated using the program Sequence Matrix v1.7.8 (Vaidya, Lohman, & Meier, 2011). Multiple‐sequence alignments were conducted using the program Bioedit version 7.1.3.0 (Hall, 1999) with default settings. The concatenated sequences were format converted for further analysis using the Geneious v9.1.4 program. Subsequently, a concatenated data were tested using partition homogeneity test as implemented in PAUP* v.4b.10 (Farris, Källersjö, Kluge, & Bult, 1994; Swofford, 2003), and the result of the sequences incongruence test was not significant (*p* = 0.956 > 0.05) and indicated that five genes can be concatenated together. The program DnaSP v5 (Librado & Rozas, 2009) was used to identify haplotypes and counted polymorphic sites of *C. kamensis* DNA sequences. Phylogenetic analyses were conducted with the program MrBayes 3.2.6 (Ronquist et al., 2012) using a Bayesian inference (BI) approach (Yang & Rannala, 1997). Here, *P. roborovskii* and *P. sungorus* (Cricetinae, *Phodopus*) were used as outgroups (Lebedev et al., 2018). The model with the best fit of nucleotide substitution and the best partition schemes for the DNA dataset was identified under the Corrected Akaike Information Criterion (AICc) (Akaike, 1974), which was implemented in the MrModeltest 3.7 program (Posada & Crandall, 1998). Model test result showed that the best‐fitted substitution model for the concatenated data was GTR + I + G. Bayesian analysis was conducted using MrBayes 3.2.6, which was run with twenty million generations of Markov Chain Monte Carlo (MCMC) simulation, and sampled every 1,000 generations. MCMC was run using the default model parameters, starting from a random tree. The first 25% were discarded as a conservative burn‐in, and the remaining samples were used to generate a 50% majority rule consensus tree. Here, a Bayesian posterior probability equal or above 0.95 was considered to indicate strong relationships (Leaché & Reeder, 2002). In addition, to represent relationships between haplotypes, the program Network 4.6.0.0 (Bandelt, Forster, & Rohl, 1999) was run to construct clustered relationships for the concatenated mtDNA haplotypes of *C. kamensis* with a median‐joining network approach.

### 2.4 Nucleotide mutation rate and estimation of divergence time

To acquire as accurate divergence time as possible, we estimated the number of nucleotide substitutions per site (d) from comparisons of the focal species and an outgroup species using the formula: d = (tv + tvR)/m, where tv is the number of transversions between the *C. kamensis* and outgroup taxa, R is the transition/transversion ratio within the *C. kamensis* complex, m is the sequence length (Nei, 1992; Rooney, Honeycutt, & Derr, 2001). Transition and transversion values were calculated in the program MEGA 6.0 (Tamura, Stecher, Peterson, Filipski, & Kumar, 2013). The rate of nucleotide substitutions per site per lineage per year is λ = d/2T when an estimate of d was obtained, where T is the divergence time between the ingroup and outgroup species (Rooney et al., 2001). The mutation rate per nucleotide site per generation is μ = λg, where g is the generation time (g = 1/2 year of the *C. kamensis* complex) (Luo et al., 2000). Here, d was 0.058 (tv = 43, R = 5.82, m = 5,078) for combined genes (nuclear and mitochondrial genes). The rate of nucleotide substitution per site per lineage per year (λ) was about 0.278 × 10^−8^ (T = 10.37 Mya, quoted from Lebedev et al. (2018)), and the mutation rate of per generation (μ) was about 0.139% Mya for concatenation DNA sequence of *C. kamensis*.

Divergence times among the different populations of *C. kamensis* were estimated using BEAST version 1.7.4 (Drummond & Rambaut, 2007). There were 5,078 bp in the concatenated genes (mtDNA + nuDNA) within the *C. kamensis* complex. Phylogenetic relationships were reconstructed with the Yule speciation process (Steel & McKenzie, 2001), with enforced monophyly of the ingroup. MCMC analysis was run twice, chain lengths were 50 million, sampling every 1,000 generations, and the first 10% were discarded as burn‐in. One calibration point was used as prior for the time for most recent common ancestor of *P. roborovskii* and *P. sungorus*. The age of the *P. roborovskii*/*P. sungorus* divergence (5.69 Mya) was used as calibration interval (Lebedev et al., 2018). Here, a normal distribution was chosen as the prior model for the calibration with mean 5.69 Mya and standard deviation 0.01 (a central 95% range of about 5.67–5.71 Mya). The final chronogram was generated with the program TreeAnnotator 1.7.4 (a subprogram of BEAST) using the mean time as node height. The program Tracer v1.5 was used to test the validity of the sampling result of MrBayes and BEAST (effective sample size (ESS)>200). Finally, all tree files were visualized using the FigTree v. 1.3.1 program.

### 2.5 Population genetic structure and divergence

Population genetic parameters were calculated for combined mtDNA genes. Haplotypes were assigned to groups based on the phylogenetic relationships between geographic populations. The general parameters of diversity in the combined dataset were calculated for each clade using the program DnaSP v5, including numbers of haplotypes (H) and unique haplotypes (UH), haplotype diversity (Hd), nucleotide diversity (π), and the average number of pairwise nucleotide differences (K). The percentages of unique haplotypes per population were calculated via unique haplotypes divided by the number of haplotypes in a given population. Here, three sample sites were removed because only one sample was available or the nucleotide diversity was zero (Table 1). Analysis of molecular variance (AMOVA) was conducted using the program Arlequin version 3.5 (Excoffier, Laval, & Schneider, 2007) to identify variation among individuals, populations, and clades, and the significance was tested by 10,000 permutations. The derived populations were grouped based on the phylogeographic structure obtained in the phylogenetic analyses. The population differentiation indexes (*F_ST_*) (Hudson, Boos, & Kaplan, 1992b) between populations were calculated in Arlequin and gene flow (Nm) between populations was calculated using DNASP (Hudson, Slatkin, & Maddison, 1992a; Librado & Rozas, 2009). The genetic distance between observed clades was calculated using MEGA 6 (Tamura et al., 2013) with the Kimura‐2‐parameter (K2P) model of nucleotide substitution (Kimura, 1980).

### 2.6 Demographic history

Three methods were used in demographic analyses of *C. kamensis*. Potential signals of population growth were detected using *Fu's FS* (Fu, 1997), *Tajima's D* (Tajima, 1989), and R2 (RamosOnsins & Rozas, 2002) under a model of sudden expansion, and these parameters were calculated in the program Arlequin and DnaSP. Furthermore, the pairwise mismatch distribution of the combined sequences (mtDNA) was computed in DnaSP to identify the potential signal of demographic expansion of the major clades. A goodness‐of‐fit test was used to investigate whether the observed data fitted a model of recent expansion (Slatkin & Hudson, 1991). In addition, the raggedness indices (r) were calculated in DnaSP to determine whether the concatenated dataset deviated significantly from the population expansion model (Harpending, 1994). A Bayesian skyline plot (BSP) was implemented in BEAST to estimate the effective population size over time (Heled & Drummond, 2008). Here, a strict clock was used in BEAST using an average substitution rate according to the estimated results of nucleotide mutation, as described above. The same nucleotide substitution model was used for Bayesian phylogenetic analysis. The BSP was run twice for MCMC chain lengths of 50 million generations, sampled every 1,000 generations, and both the MCMC convergence and the ESS were tested with the program Tracer v1.5.

### 2.7 Species distribution modeling

The species distribution model (SDM) of suitable habitats for *C. kamensis* was implemented in the Maxent v. 3.3.3 program using maximum entropy machine‐learning algorithm fluctuations (Phillips, Anderson, & Schapire, 2006; Phillips & Dudík, 2008). The climate dataset was downloaded from the WorldClim v1.4 database (www.worldclim.org), the layers contained four periods: the last interglacial (LIG; about 120,000–140,000 years ago (120–140 Ka)), the last glacial maximum (LGM; about 21 Ka), the Mid‐Holocene (MH; about 6 Ka), and the present (Current; 1960–1990). Among these, the paleoclimate data were downloaded from the community climate system model (CCSM) with 30 arc‐second and 2.5 arc‐minute resolutions (Hijmans, Cameron, Parra, Jones, & Jarvis, 2005; Otto‐Bliesner, Marshall, Overpeck, Miller, & Hu, 2006), respectively. For the SDM analysis, a climate dataset was used with a subset of 19 bioclimatic variables at 2.5 arc‐minute (about 4 kilometers) spatial resolution, and a 30 arc‐second resolution was resampled into a 2.5 arc‐minute resolution using ArcGIS 10.3 (ESRI). These variables were extracted by the “Mask” method at a range of 26° to 41°N and 77° to 104°E region. A total of 27 occurrence records of *C. kamensis* were obtained from our field records, literatures, and the GBIF web site (www.gbif.org). To eliminate a potential overfitting effect, the percent contribution between 19 bioclimatic variables were tested using the ENMeval package in R language (Muscarella et al., 2014; R Core Team, 2017). Moreover, the regularization multiplier value was set up with a range of 2–4, which significantly reduced the difference between calibration AUC (area under curve, AUC), evaluation AUC, and omission rate without affecting the evaluation AUC (Radosavljevic, Anderson, & Araújo, 2014). SDM analysis was run in Maxent, and the data were randomly split into two subsets in which the 80% occurrence data was used as training data and the remaining 20% were used as testing data. The model used the default convergence threshold, 5,000 maximum iterations, and 10 replications. A receiver operating characteristic curve close to 1 indicated better model performance (Phillips et al., 2006).

## 3 RESULTS

### 3.1 Phylogeographic analysis and population divergence time

**Figure 2:**
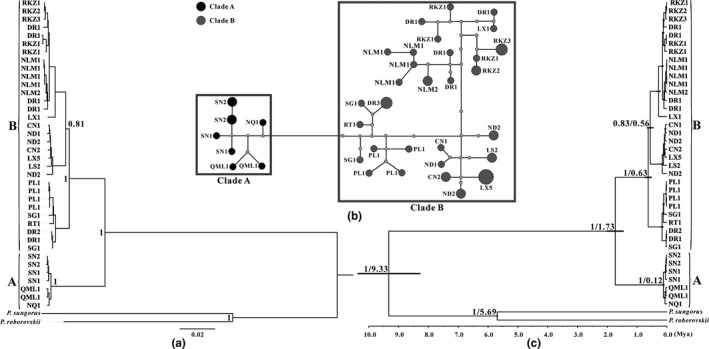
Bayesian tree, haplotype network and chronogram are estimated by the haplotypes of *C. kamensis*. (a) Consensus tree constructed with the concatenated haplotypes (mtDNA + nuDNA) of *C. kamensis* using Bayesian inference. The posterior probabilities are indicated at the major inner nodes, and population names are provided in Table 1. The two major lineages are denoted by A (from the east and north of the QTP) and B (from the south and southwest of the QTP). (b) Median‐joining network of *C. kamensis* infer from the combined mtDNA haplotypes. Circle sizes are proportional to the number of individuals that share the same haplotypes. The missing haplotypes in the network are represented by gray dots. The circle name is the haplotype of different locations and the number of shared haplotypes. Evolutionary clades correspond to the two major clades in Figure 2a. (c) A chronogram of *C. kamensis* is estimated by the concatenated haplotypes using the Bayesian strict clock (using the estimated mutation rate) in BEAST. Branch lengths represent the mean values of the posterior distribution. The node bars indicate the posterior probability distribution of the node age under the 95% CI. The posterior probability value and divergence time are indicated at each node. The two major lineages are denoted by A and B in line with the Bayesian tree

The phylogenetic tree showed two major evolutionary clades (A and B) with high nodal posterior probability (PP) values (PP = 1) inferred from DNA concatenated sequences (Figure 2a). The A clade indicated that the Qilian Mountains population (SN) have a close relationship with the Bayan har Mountains populations (NQ and QML). Moreover, the B clade was involved in all geographic populations from the Tibet region (Himalaya Mountains) with a strong support value (Figure 2a; sample information is presented in Table 1 and Figure 1). In addition, median‐joining network analysis also indicated the divergence of two major clades, which was consistent with the Bayesian phylogenetic tree (Figure 2b). In conclusion, phylogenetic topologies indicated strong geographic variations between these two clades.

Divergence time analysis also resulted in two major clades that represented two derived populations in the species tree with the same topological structure, corresponding to Bayesian tree (Figure 2c). As such, the species tree supported these two major clades (A and B) with high support value (PP = 1) in the Bayesian dating analysis. The divergence time between A and B lineages dated back to the Early Pleistocene (about 1.73 Mya) for the concatenated data with 95% confidence intervals (95% CI) of 1.46 to 2.03 Mya.

### 3.2 Population genetic structure

A total of 53 individuals generated 37 haplotypes, which considered alignment gaps and invariable sites based on concatenated mtDNA sequences. Most *C. kamensis* populations had a higher UH ratio among populations except for the LS population (Table 1). Twenty‐six unique haplotypes were found in 12 populations, indicating high levels of genetic variation among *C. kamensis* populations. The average number of nucleotide differences (K) between geographic populations indicated that population DR had a higher *K* value (*K* = 68.571) and showed fine DNA heterogeneity (Table 1). The nucleotide diversities (π) of all 13 populations were quite low (π = 0.001–0.016), and the DR population was the highest (π = 0.016) compared to other populations (Table 1). To reduce the small sample bias, adjacent population were merged with few samples into a bigger number population depending on phylogeographic structure, including two major lineages A and B (Table 3 and Figure 2). The Hd was high (0.944–0.976) between Clades A and B, while the π was 0.003 and 0.017, respectively. Pairwise *F_ST_* values indicated that the geographic populations from East‐North QTP were significantly divergent form all other geographic populations from West‐QTP and South‐QTP and the overall *F_ST_* value of Clades A and B was 0.776 (*p* < 0.01). AMOVA analysis resulted in a significant pattern of variation within two clades (75.04%) based on concatenated mtDNA sequences, and the variance between sampled localities within clades and within sampled localities was 17.82% and 7.14% (Table 4), respectively. Likewise, significant differentiation (88.25%) was also found between Clades A and B inferred from vWF gene, and the variance between sampled localities within clades and within sampled localities was 3.99% and 7.76% (Table 4), respectively.

**Table 3:** Genetic diversity indices, neutrality test results, and demographic analysis for each population of *C. kamensis*

Parameter	Clade A	Clade B
N	9	44
H	7	29
Hd	0.944	0.976
π	0.003	0.017
K	12.722	72.450
*Fu's Fs*	0.783	3.487
*Tajima's D*	−0.200	0.515
R2	0.136	0.127
r	0.049	0.009
Mismatch distribution	Multimodal	Multimodal

Note

Raggedness statistic (*r*), Ramos‐Onsins and Rozas (R2).

**Table 4:** Results of hierarchical AMOVA of genetic variations of *C. kamensis* populations

Locus	Source of variation	*df*	Sum of squares	Variance components	Percentage of variation	Fixation indices
mtDNA	Among two clades	1	1766.772	109.329**	75.04	FCT = 0.750**
	Among populations within clades	11	1228.109	25.962**	17.82	FSC = 0.714**
	Within populations	40	415.909	10.398**	7.14	FST = 0.929**
vWF	Among two clades	1	9.823	0.644**	88.25	FCT = 0.883**
	Among populations within clades	11	1.872	0.029*	3.99	FSC = 0.340*
	Within populations	40	2.265	0.057**	7.76	FST = 0.922**

Note

Populations were grouped into two subsets that correspond to reciprocally monophyletic clades.

* Significant at *p* < 0.05.

** Significant at *p* < 0.01.

### 3.3 Gene flow and K2P distance

The gene flow was low (Nm = 0.334 for concatenated mtDNA; Nm = 0.289 for vWF gene), and significant genetic differentiation (*F_ST_* = 0.776 for concatenated mtDNA, *p* < 0.05; *F_ST_* = 0.889 for vWF gene, *p* < 0.05) was found between A and B lineages in the QTP. Moreover, pairwise *F_ST_* values were high and significant, which indicated restricted gene flow between two recovered populations. Furthermore, the K2P distance was 0.064 between Clades A and B based on concatenated mtDNA haplotypes, was 0.067 for CYTB haplotypes and was 0.003 for vWF haplotypes.

### 3.4 Demographic history

**Figure 3:**
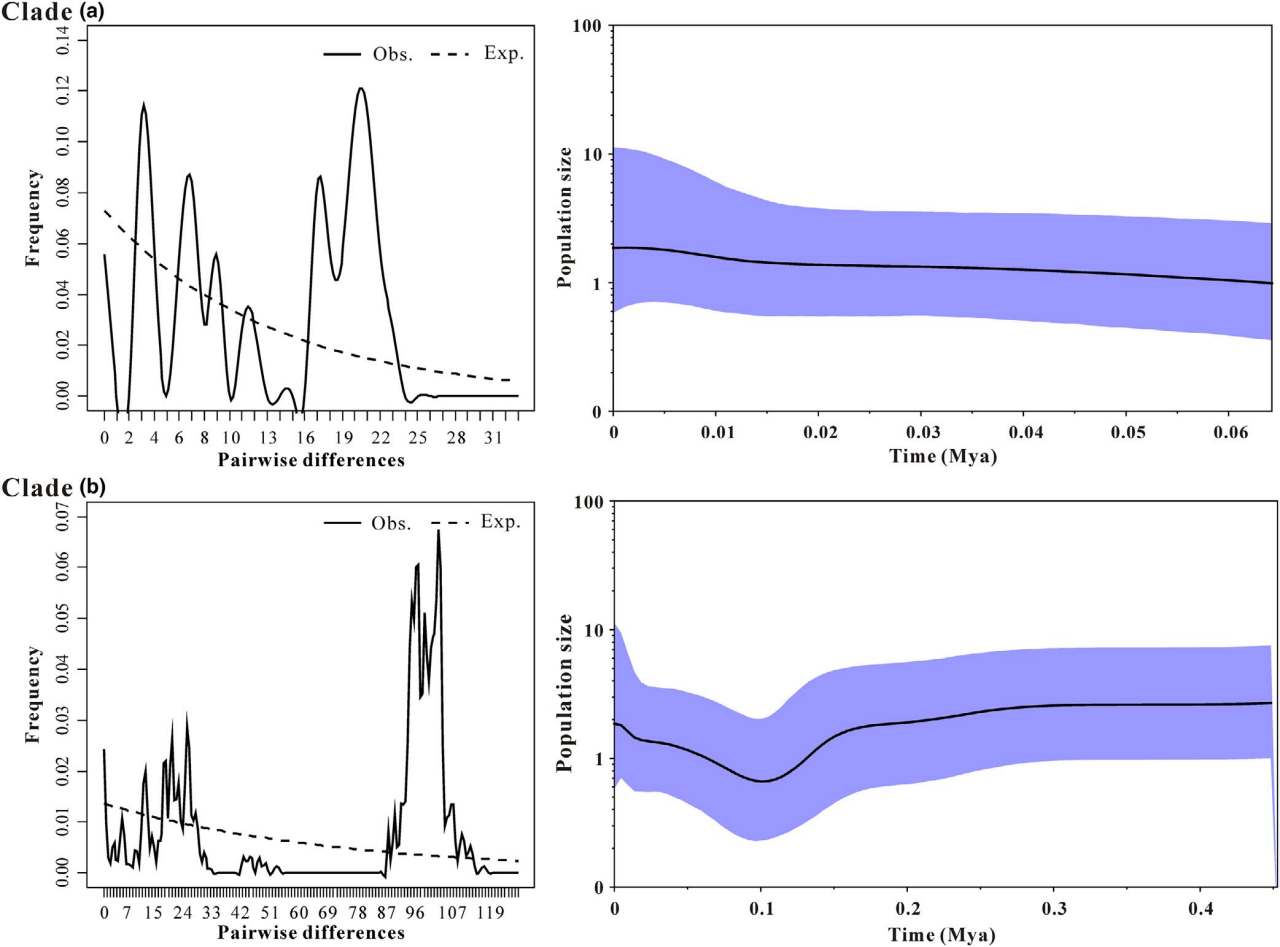
Demographic history of *C. kamensis* infers from the concatenated mtDNA haplotypes. (a) Diagrams of mismatch distributions for two major lineages. The solid line indicates the observed frequency of pairwise nucleotide differences between sequences, and the dashed line indicates the expected distribution based on a model of sudden population expansion. (b) Bayesian skyline plots for two major clades of *C. kamensis*. The black line represents the median estimated population size, and the blue‐shaded areas represent the 95% confidence interval of HPD analysis in the BSP

Neutral tests indicated that *Tajima's D* values were negative and no significant difference (*p* > 0.05) in lineages A and B were found. They exhibited an observed multimodal mismatch frequency distribution (Figure 3a), suggesting past population did not go through sudden expansion, but instead accepted the constant size model. *Fu's Fs* were detected in both A and B lineages, and were positive, with neither significant nor unimodal distribution. Those suggest that the past population showed no explicit signals of population expansion or equilibrium (Figure 3a). Moreover, the R2 value was not significant for two derived populations, which also supported the constant expansion model (Table 3). For the mismatch distribution analysis, both A and B lineages showed an observed multimodal mismatch frequency distribution (Figure 3a), in contrast to a sudden population expansion model. In addition, the demographic history of *C. kamensis* was also indicated by the BSP analysis for the same derived two populations (Figure 3b). According to the BSP results, the population size of Clade B followed a small increase after a prolonged period of slow decrease throughout history (time node: 0.10 Mya) (Figure 3b), whereas Clade A showed a constant population size trend over time.

### 3.5 Species distribution modeling

**Figure 4:**
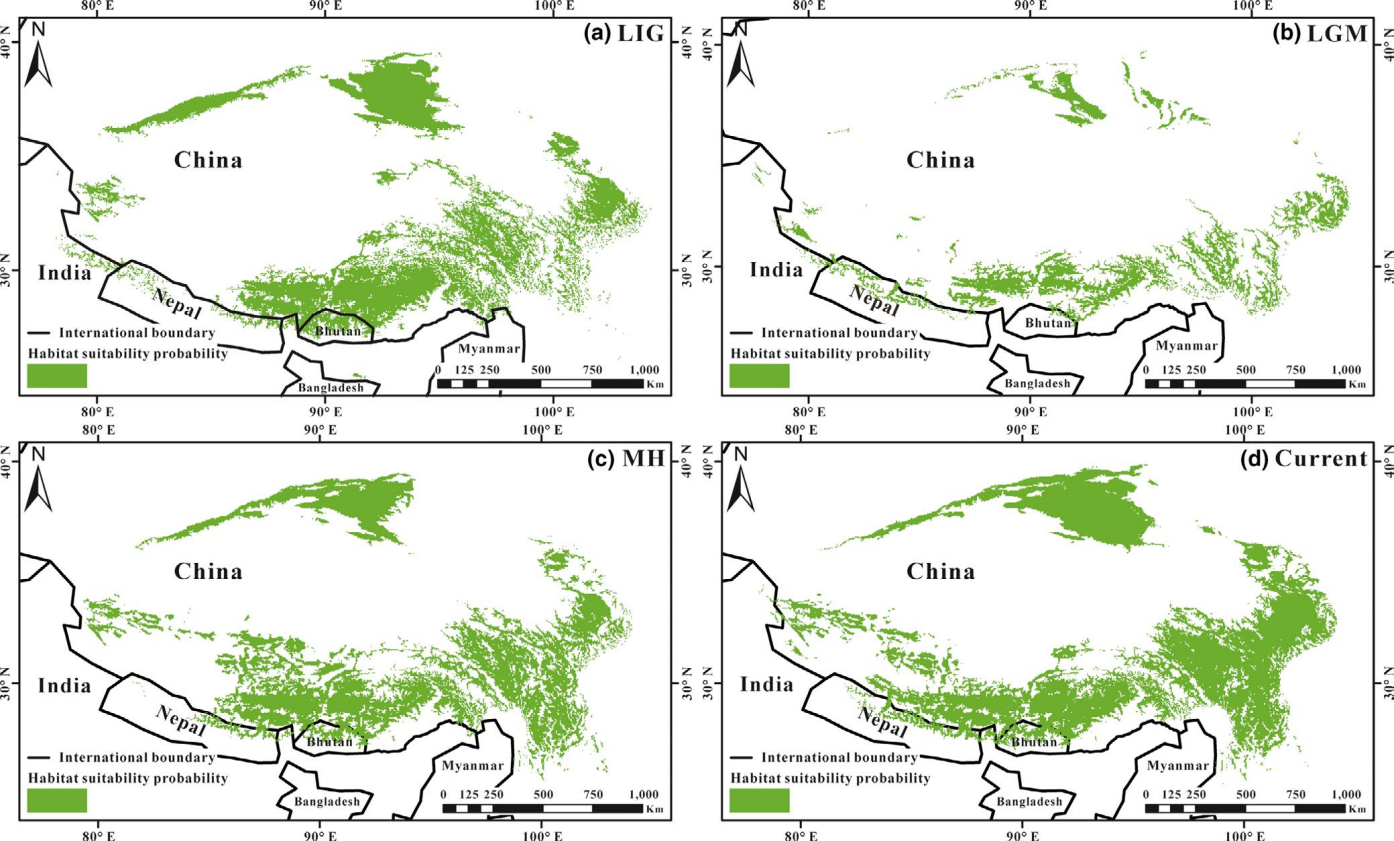
Species distribution model of suitable habitats for *C. kamensis* estimated via Maxent. The estimates are inferred from the Last Interglacial (LIG), the Last Glacial Maximum (LGM), the Mid‐Holocene (MH), and the current climate (Current). The green area represents the suitable habitat for *C. kamensis*

The SDM analysis showed an optimal prediction of the *C. kamensis* distribution with high AUC value (LIG, 0.934; LGM, 0.956; MH, 0.901; Current, 0.925). SDM showed that the suitable habitat was the most widespread during the LIG (Figure 4a). It is worth noting that the Arjin Mountains provided a suitable habitat for the survival of *C. kamensis* during the LIG, and then, the habitat gradually diminished. The results of comparisons with other three periods indicate that the potential distributions of *C. kamensis* were most restricted and shifted toward at lower elevations on the edge and valleys of the QTP during the LGM (Figure 4b). After the LGM, the suitable habitat was restored to a certain extent based on the potential distribution of MH for *C. kamensis*. Compared to the MH, the current distribution of *C. kamensis* was increased, especially in the east and north of the QTP increased (Figure 4c and d).

## 4 DISCUSSION

### 4.1 Phylogeographic structure: QTP uplift and isolation

The phylogenetic results showed that the haplotypes of *C. kamensis* from different geographic populations split into two distinct clades (Clades A and B, PP = 1) inferred from the BI and BEAST. Moreover, the Bayesian tree and median‐joining network resulted in the same topological structure (Figure 2a and b). The results of the molecular clock indicate that the divergence between Clades A and B occurred in the Early Pleistocene (about 1.73 Mya, 95% CI: 1.46–2.03 Mya) (Figure 2c). For this scenario, several factors caused an intraspecific phylogeographic pattern in organisms, including abiotic and biotic factors, such as geography, climate, habitat preference, and dispersal ability (Fan et al., 2012; Qu, Lei, Zhang, & Lu, 2010; Wang et al., 2014). Here, the time of the differentiation of Clades A and B coincides with the active period of the Late Cenozoic QTP. The QTP had risen to the present elevation during the Late Cenozoic after several severe uplifting events. The Qing‐Zang tectonic movement occurred from the Middle Pliocene to the Early Pleistocene (between 3.4 and 1.7 Mya), which contains three phases A (began in 3.4 Mya), B (2.5 Mya), and C (1.7 Mya) (Shi et al., 1999). Two major population divergences coincided with the C phase, namely the Qing‐Zang tectonic movement induced population differentiation between Clades A and B. Moreover, several geographic barriers formed by tectonic movements, such as mountains, rivers, deserts, and permanent tundra, restrict gene exchange among populations for *C. kamensis*. It is worth noting that the Tanggula Mountains showed an intensive uplift event at the Middle Pliocene (about 3.7 Mya) due to the east‐west extension of the Eurasian plate after the collision, which reaches very high elevations and was sufficient to block the colonization of animal with low dispersal abilities such as rodents (Duan, 2005; Jin & Liu, 2010). In addition, a stable isotope study indicated that the Kunlun Mountains reached considerable elevation during the Late Pliocene (about 4,700 a.s.l.) (Wang et al., 2008b), which also blocked the gene exchange between *C. kamensis* populations. Furthermore, the rapid uplift of the Nyenchentanglha Mountains also occurred during the Middle Pliocene (about 3.7 Mya) (Wu, Liu, Hu, & Ye, 2003) and also formed a major geographic barrier that impeded the genetic exchange between Clades A and B (Zhu, Zhenhan, Zhao, Jianping, & Wang, 2012).

Prior to the Qing‐Zang tectonic movement (3.4 Mya), this region had a typical subtropical climate and a large number of mammalian fossils can be found in the QTP, for example, of *Hipparion chilongensis*, *Coelodonta thibetana*, and *Panthera blytheae*, which confirm climatic assumptions (Deng & Ding, 2015; Deng et al., 2015; Li et al., 1979). At relatively low altitude, animals can freely roam the plateau and lowland, and at that time, numerous animals obligately inhabited a warm and humid environment (Deng, 2016). Theoretically, there are wide areas on the plateau that was suitable for the survival of *C. kamensis* at that time, rather than around the QTP, like the current distribution (Feng et al., 1986; Luo et al., 2000). Three viewpoints can explain the current phylogeographical structure of *C. kamensis*. First, mountains and rivers were formed by the Qing‐Zang tectonic movements, which created geographic barriers that reduced the gene flow between isolated populations and promoted allopatric divergence, which often restricted animal dispersal, particularly that of small mammals with weak diffusion capacity such as rodents (Wang et al., 2014). For instance, Fan et al. (2012) suggested that the major clades for the differentiation of *Apodemus draco* that occurred during the Late Pliocene to Middle Pleistocene correspond to the Qing‐Zang tectonic movements and subsequent geological events. Second, the Kunlun‐Huanghe tectonic movement in the Middle Pleistocene (between 1.1 and 0.6 Mya) caused the average altitude of the QTP to exceed 3,000 m (a.s.l.) after the Qing‐Zang tectonic movement, and a large area of the plateau entered the frozen circle (Li & Fang, 1999; Shi, Zheng, Li, & Ye, 1995). The subsequent Gonghe tectonic movement raised the QTP as a whole to the current altitude during the Late Pleistocene (about 0.15 Mya) (Li & Fang, 1999). As a result of the blocking of the northward flow of the Indian Ocean warm current and the strengthening of the winter monsoon, caused drier and colder climate, especially for the northern plateau (An et al., 2006; Lei et al., 2014; Wu et al., 2001). This change of climate caused a dramatic ecological shift in the QTP, where forests were replaced by grasslands, glaciers started to develop, and both deserts and permafrost areas formed (Wu et al., 2001). In addition, the uplift of the QTP resulted in shaping the paleolake in the Qaidam Basin during the Late Pliocene (about 2.5 Mya), and a growing winter monsoon caused the shrinkage and disappearance of ancient lakes and an evolution of desertification (Wang, Huang, & Liu, 2002; Wang et al., 2009; Zeng, Feng, & Cao, 2003). Moreover, the uplift of the QTP also promoted aridification and the development of vast deserts in the Gonghe basin (Liu, Wang, Wang, Hideaki, & Abbott, 2012). Consequently, mountains uplift and aridification promoted the divergence between the Qilian Mountains population and the Bayan har Mountains population. Third, the uplift of the QTP induced changes of plant communities, which can affect the distribution of *C. kamensis* (Jia et al., 2012; Liu, Wang, Wang, Hideaki, & Abbott, 2006). These environmental factors caused the favorable habitat of *C. kamensis* to shrink to margin regions or intermountain basins with relatively low altitude in the QTP. *C. kamensis* show a strong habitat preference, which makes them highly sensitive to environmental change. Consequently, it was easy to understand that the uplift of the QTP caused isolation among populations. A lack of gene exchange between populations let each population evolve in its own direction. In summary, all of these rational factors can lead to the current phylogeographical pattern of *C. kamensis*.

### 4.2 Genetic structure and divergence of the *C. kamensis* population

All total of 53 *C. kamensis* specimens were collected from 13 geographic populations in the QTP, all of which were part of two recovered populations and were subjected to population genetic analysis. It is worth noting that there was no shared haplotype among populations, and each population had higher UH ratio, and individuals in the population had higher nucleotide differences (*K* value, see Table 1). Moreover, the recovered populations showed similar results based on the phylogeographical patterns of *C. kamensis* (Table 3). Here, 13 geographic populations and two derived populations showed low genetic diversity (Tables 1 and 3). AMOVA indicated highly restricted gene flow and fragmented divergence throughout the distributional range of the species (Table 4). The QTP features numerous mountains, glaciers, rivers, lakes, deserts, and permafrost regions, and these abiotic factors can block the colonization and gene exchange of small mammals such as rodents (Nava‐García et al., 2016). At this point, the available data enables the understanding of why the *C. kamensis* population does not have shared haplotypes. More importantly, the high *F_ST_*, low Nm, higher K2P value, and genetic variation among populations confirmed this point. In addition, life history factor may contribute to this genetic pattern. *C. kamensis* preferred to live in valleys or riversides with leguminous or chenopodiaceous plants (Luo et al., 2000), and SDM analysis further validated its habitat preferences. Thus, geographic barriers restrict the gene exchange between populations, such as *Nanorana pleskei* (Dicroglossidae) (Zhou et al., 2017). The reason for the high UH ratio and nucleotide differences in *C. kamensis* populations was that its population density is comparatively small and it has been listed as near threatened in a recent survey (Jiang et al., 2016). The *C. kamensis* populations showed low genetic diversity (Tables 1 and 3), which was related to the harsh environment on the QTP. Jin and Liu (2008) suggested that organisms living at high‐altitude areas with high selective pressure develop a specific genotype for survival. In contrast, suitable environments with relaxed selective pressure can accommodate more genotypes and the population possesses higher genetic diversity (Jin & Liu, 2008), such as *Phrynocephalus vlangalii*. Thus, the genetic structure of the *C. kamensis* population is strongly affected by the harsh environment of the QTP.

### 4.3 Demographic history: Glacial oscillations and population colonization

Neutrality tests for the derived populations indicated that *Fu's FS*, *Tajima's D*, and R2 were not significantly negative, and mismatch distributions were multimodal rather than following a Poisson distribution, suggesting that two recovered populations did not go through a sudden expansion in history (Table 3; Figure 3). BSP analysis indicated that the population size fluctuating slightly at the Late Pleistocene (about 0.10 Mya) and showed an increase in population since then (Figure 3). The reason why the *C. kamensis* population had not experienced rapid expansion lies in the complicated landform configuration and the harsh environment after the uplift of the QTP. Both extrinsic and intrinsic factors interacted to generate those results. Intrinsic factors include that the population density of *C. kamensis* is low and that they are not the dominant species on the QTP (Feng et al., 1986; Jiang et al., 2016). This hamster prefers to live alone and has a lower reproductive capacity in comparison with gregarious rodents such as *Merions meridianus* (Rodentia, Gerbillinae) (Luo et al., 2000). Furthermore, its habitat preference and weaker locomotion were also potential intrinsic factors. Extrinsic factors were that the uplift of the QTP resulted in a decrease in suitable habitat of *C. kamensis*. The species was restricted to microhabitats and dispersed in valleys and riversides of the QTP (Figure 4). At this point, the Quaternary glacial period exerted little effect on the population size of *C. kamensis* (Figure 3) because valleys or riversides are generally at lower altitudes and can temporarily help animals through the harsh environment (Fan et al., 2012; Qu et al., 2010). The last glacial period only caused a fluctuation in the *C. kamensis* population size during the Late Pleistocene (Zhu et al., 2012), which was confirmed by BSP analyses (Figure 3). Areas of low altitude (e.g., valleys, riversides, and intermontane basins) are not covered by ice sheets and can thus provide significant suitable microhabitats for organisms during the glacial period (Zheng & Rutter, 1998). A variety of vertebrates in the QTP benefit from glacial refugia, and the populations began to expand after the end of the glacial period, such as *Montifringilla adamsi* and *Pyrgilauda blanfordi* (Qu et al., 2010), *A. draco* (Fan et al., 2012), and *N. parkeri* (Liu et al., 2015). Therefore, *C. kamensis* survived the Late Pleistocene glacial period.

The results of SDM analyses indicate that the LGM induced a significant decrease in the *C. kamensis* suitable habitat in comparison with the LIG (Figure 4). The LGM was larger than the previous glacial period so that suitable habitats of *C. kamensis* may have been insufficient for their survival (Shi et al., 1998). Gupta, Sharma, & Shah (1992) and Zheng and Rutter (1998) suggested that the whole plateau is covered by a huge ice sheet during the glacial period, and force most species to retreat to favorable habitat at lower elevations on the edge of the plateau during glacial maxima, from which they recolonized the interior during interglacials (Fan et al., 2012; Lei et al., 2014; Tang et al., 2010). Several recent phylogeographic studies support this hypothesis, SDM analysis showed that suitable habitats of *N. pleskei* were also significantly reduced during the LGM (Liu et al., 2015; Wang et al., 2017). Later, suitable habitats for *C. kamensis* survival were restored since the Mid‐Holocene, and the suitable habitat was able to expand again (Figure 4). The reason was that the warm and humid climate on the QTP was suitable for animals and plants to thrive during the Mid‐Holocene, suitable habitats may have increased since the LGM, and consequently, organisms living at the QTP could expand (Shi et al., 1998). In addition, the precipitation in the east of the plateau has decreased from the southeast to the northwest, since a region with high precipitation is located in eastern Tibet and the western Sichuan plateau, while a region with low precipitation is located in the Qaidam Basin (Hu & Liang, 2013). Theoretically, within a certain range of precipitation, the diversity of rodents correlates positively with precipitation (Abramsky & Rosenzweig, 1984; Ye, Ma, & Feng, 1998). A case study suggested that small mammals from the semiarid environment are more likely to choose a habitat with higher precipitation (Milstead et al., 2007). This explained why the suitable habitat of *C. kamensis* increased in the east of the QTP under the current climatic conditions.

### 4.4 Review on the *C. kamensis* classification

The classification of Tibetan hamster has been controversial over the past century. Historically, Satunin named the female hamster from the southeastern part of the Qinghai–Tibet Plateau for the first time as an *Urocricetus kamensis* (Type locality: the Moktschjun River in the upper reaches of the Lantsang River) in 1902 and named the specimen with a pale pelage from Qilian Mountain as the *C. kozlovi* (Satunin, 1902). Then, Bonhote named a specimen from Lhasa (Tibet autonomous region, China) as a *C. lala* in 1905 (Bonhote, 1905), and Thomas named the specimen with shorter tail and pale pelage from the Ladakh region adjacent to the northern Qinghai–Tibet Plateau as the *C. alticola* (Thomas, 1917). Consequently, Tibetan hamster was classified as four separate hamsters (*U. kamensis*, *C. kozlovi*, *C. kama,* and *C. alticola*). As such, Smith and Xie (2013) identified four hamsters in the QTP based on morphological characteristics, namely *C. alticola*, *C. kamensis*, *C. lama,* and *C. tibetanus*. Argyropulo (1933) sorted the Tibetan hamster specimens and divided it into three species (*C. kamensis*, *C. kozlovi* and *C. lama*) in 1933. However, Corbet (1978) admitted that *C. kamensis* and *C. alticola* are valid species, and the other three species (*C. kozlovi*, *C. lama* and *C. tibetanus)* are synonyms of *C. kamensis* (Corbet, 1978). Moreover, Don and DeeAnn (2005) accepted Corbet (1978)’s opinion that both *C. kamensis* and *C. alticola* are valid species. Although they have a detailed description of morphology of Tibetan hamster, unfortunately, a small sample size can cause errors in its classification (Mayr, Linsley, & Usinger, 1953), such as age variation, seasonal variation, and ecological variation. Wang et al. (1973) pointed out *C. alticola* is likely to a subspecies of *C. kamensis*, and then Zheng (1979) confirmed that this conclusion is correct in the scientific investigation of animals and plants in the Ali area of Tibet (China). Feng et al. (1986) and Luo et al. (2000) also suggested that *C. alticola* is a subspecies of *C. kamensis* rather than a valid species. In conclusion, four hamsters (*C. kozlovi*, *C. lama*, *C. alticola,* and *C. tibetanus*) are synonyms of *C. kamensis*.

The K2P distance between clades A and B was low (6.7%). Bradley and Baker (2001) suggested that K2P values < 2% are characteristic of intraspecific variation and values > 11% are specific recognition, whereas values between 2% and 11% had a high probability of being indicative of conspecific populations or valid species. The average K2P distance of sister species was 9.55% (Bradley & Baker, 2001), suggesting that two major clades (A and B) are considered arbitrary as a species. It was worth noting that the K2P distance between hamsters was in the range of 16.7%–26.6% that was much higher than two clades (6.7%). The K2P distance between two major clades of the *M. meridianus* complex is 8.6% and does not meet interspecific divergence level, for instance, suggesting that smaller degrees of divergence tended to characterize major within‐species phylogroups (Wang et al., 2014). Our results indicated that the K2P distance between *C. kozlovi* and *C. migratorius* was 0.219 and the K2P distance from *C. kamensis* was 0.005, in addition, suggesting that *C. kozlovi* is not a subspecies of *C. migratorius* but a *C. kamensis*, which is not consistent with Lebedev and Potapova (2008)’s inferences. In summary, the K2P values did not support two clades to be regarded as a separate species and suggested the intraspecific value.

Theoretically, it appears that phenotypical changes are regarded as responding to environmental and climatic changes (Mayr et al., 1953), such as the posterior part of the skull, coat color, and teeth morphology, whereas these have long constituted the basis of rodent classification and should be validated by molecular systematic method (Chevret & Dobigny, 2005; Ge et al., 2012). Ecological specialization triggers an uncoupling of molecular and phenotypic evolution, and the departure from a phenotypic drift pattern (Qu et al., 2013; Renaud, Chevret, & Michaux, 2007), such as climate, vegetation, and diet. Thus phenotypic plasticity actually represents a fundamental component of evolutionary change, and adaptive plasticity may involve both physiological homeostasis and morphological response (Thompson, 1991). These were closely related to environmental variation. Therefore, previous taxonomic conclusions only with morphological methods did not necessarily reflect authentic phylogenetic relationships. Currently, the taxonomic approaches intend to use DNA which can be as an auxiliary criterion for identifying a species or taxon (Arctander, Arctander, Minelli, Thomas, & Vogler, 2003). Morphologically, the differences between the four Tibetan hamsters are mainly concentrated on tail length and pelage variation (Feng et al., 1986; Luo et al., 2000). These morphological characteristics may be related to habitat surrounding, such as *Rattus* species (Yom‐Tov, Yom‐Tov, & Moller, 1999). Here, the divergence time between clades A and B did not support the evolution of them into a new species (Figure 2c) because for a vertebrate to evolve into a new species to it takes at least 2 million years on average and this timeframe is too short to evolve a new species (Avise, Walker, & Johns, 1998). In addition, the genetic distance failed to the interspecific level based on the complete CYTB data. Based on our results, taken together, four hamsters were synonyms of *C. kamensis*. Further work is needed to assess morphological variation and subspecific differentiation on *C. kamensis*.

## CONFLICT OF INTEREST

The authors declare that they have no competing interests.

## AUTHOR CONTRIBUTION

JC L conceived the study; L D was responsible for obtaining and analyzing data, and wrote the manuscript with the help of JC L.

## Data Availability

DNA sequences: Genbank accessions CYTB: KX058131–KX058134, MH142269–MH142312, MK047375–MK047379; COX1: MH161606–MH161653, MK047380–MK047384; COX3: MH161654–MH161701, MK047385–MK047389; D‐loop: MH161702–MH161749, MK047390–MK047394; vWF: MH161750–MH161797, MK047395–MK047399. Outgroup taxa sequences: *P. roborovskii* KU885975 and AM000050, *P. sungorus* MH166880 and AM000049. The K2P genetic distance: *M. auratus* EU660218, *C. longicaudatus* KM067270, *C. griseus* NC_007936, *C. migratorius* KT918407, *A. eversmanni* KP231506, *T. triton* NC_013068 and *C. cricetus* MF034880.
